# Retraction for: DNMT3B decreases extracellular matrix degradation and alleviates intervertebral disc degeneration through TRPA1 methylation to inhibit the COX2/YAP axis

**DOI:** 10.18632/aging.205844

**Published:** 2024-05-31

**Authors:** Zhiqiang Luo, Yanchao Ma, Tianning Di, Bing Ma, Hongwei Li, Jiangdong An, Yonggang Wang, Haihong Zhang

**Affiliations:** 1 Department of Orthopedics, Lanzhou University Second Hospital, Lanzhou 730030, P.R. China

**Keywords:** intervertebral disc degeneration, nucleus pulposus, extracellular matrix, inflammation, DNA methyltransferase

**This article has been retracted:** Aging has completed its investigation of this paper. We found that the immunohistochemistry image in **Figure 4B,** showing expression of collagen II in second generation NP cells from IVDD rats, duplicates an immunohistochemistry image from a previously published unrelated paper [1]. The authors replied that the results of their study confirmed that DNMT3B is only weakly expressed in degenerated rat intervertebral disc tissue. However, when they recently repeated the animal part of the study, they were unable to obtain results consistent with the original study. The *in vivo* data presented in **Figure 8** “DNMT3B alleviated IVDD through TRPA1-COX2-YAP axis” were not reproducible. Consequently, all authors agreed that the article should be retracted.
